# Supplementary material for: Biallelic variants in CHCHD4 are associated with combined OXPHOS defect leading to mitochondrial disease
Source: HGG Adv. 2026 Apr 14;7(3):100615. doi: 10.1016/j.xhgg.2026.100615 (PMC13147372; doi:10.1016/j.xhgg.2026.100615)
Supplement: Document S1. Figures S1–S4, Table S1, and supplemental methods [file mmc1.pdf]

HGGA, Volume *7*

Supplemental information

Biallelic variants in *CHCHD4* are associated with combined OXPHOS defect leading to mitochondrial disease

Matthieu Mantecon, Cerina Chhuon, Kevin Roger, Ida Chiara Guerrera, Christine Bole, Patrick Nitschke, Claire-Marie Dufeu-Bérat, Margaret Ashcroft, Robert W. Taylor, Nathalie Boddaert, and Agnès Rötig

Table of contents of Supplemental data

Figure S1

Figure S2

Figure S3

Figure S4

Table S1

Table S2 (supplied as an Excel file)

Supplemental Methods

- Cell culture
- Whole exome sequencing (WES)
- Whole-cell protein extracts
- SDS-PAGE and immunoblotting
- BN-PAGE
- CHCHD4 overexpression
- Proteomics analysis

Supplemental References

Figure S1

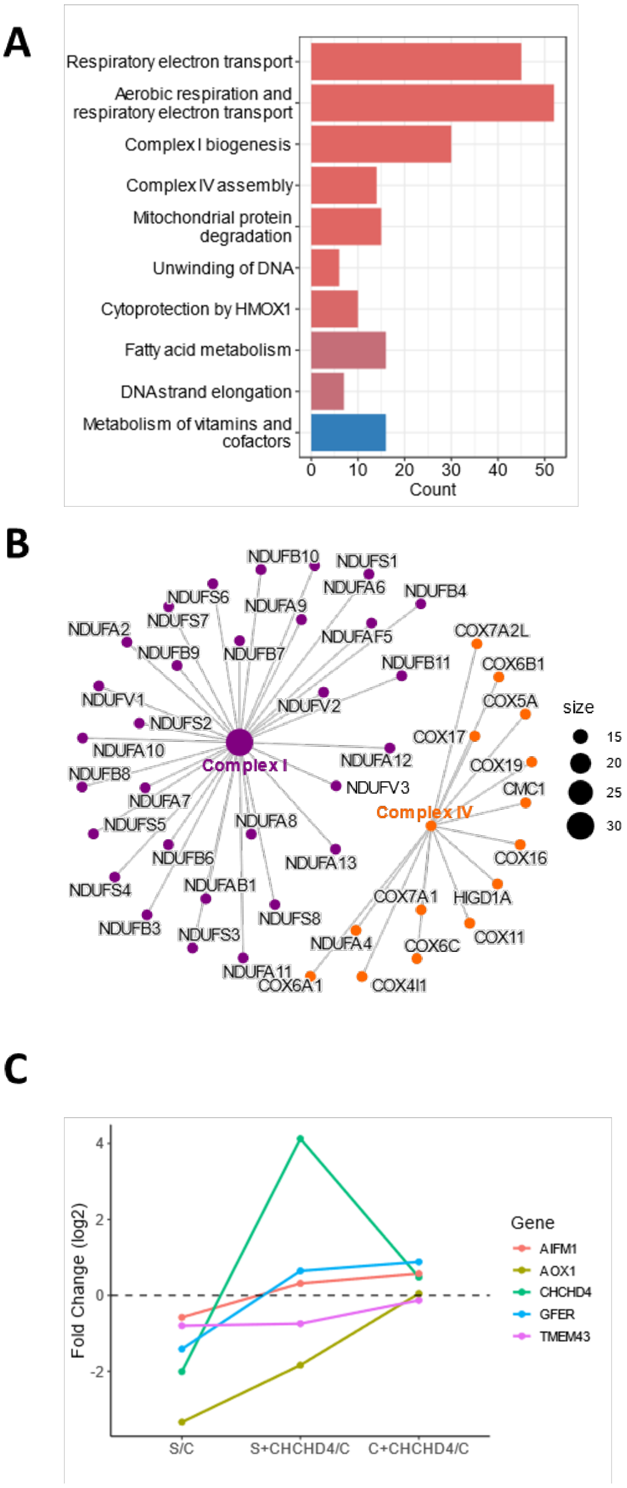

Proteomics analysis of subject (S) and control fibroblasts (C) transduced or not by wt *CHCHD4* cDNA. **A.** Reactome pathway analysis depicting the main dysregulated pathways in subject fibroblasts. **B.** Network of deregulated proteins from Complex I and Complex IV pathways in subject fibroblasts. **C.** CHCHD4 and other selected proteins levels in control fibroblasts expressed as fold change compared to control.

Figure S2

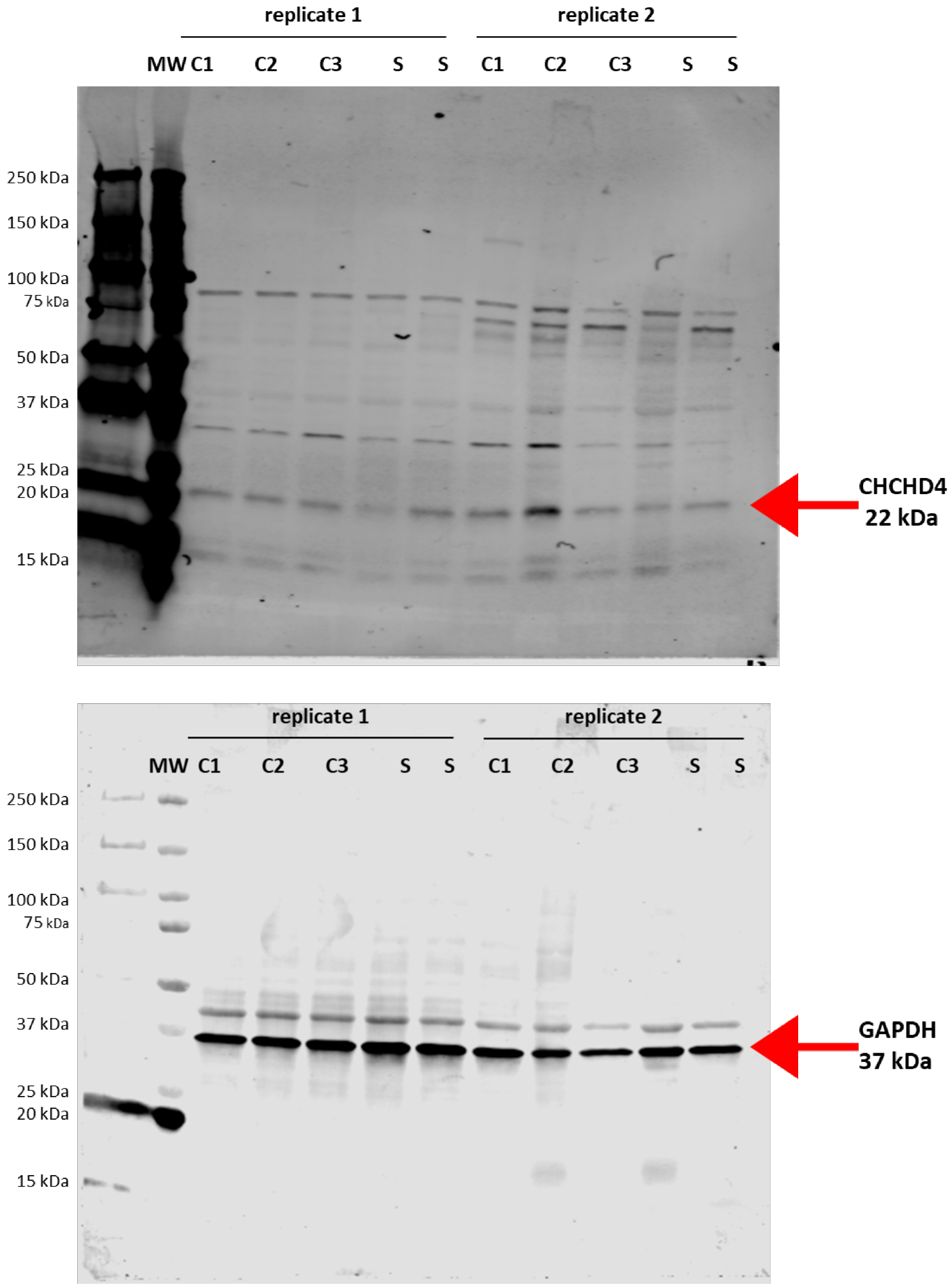

Biochemical investigation of fibroblasts. Full image of western blot analysis of CHCHD4 and GAPDH performed on total proteins of fibroblasts of subject (S) compared to 3 controls (C1, C2, C3). MW: molecular weight.

Figure S3

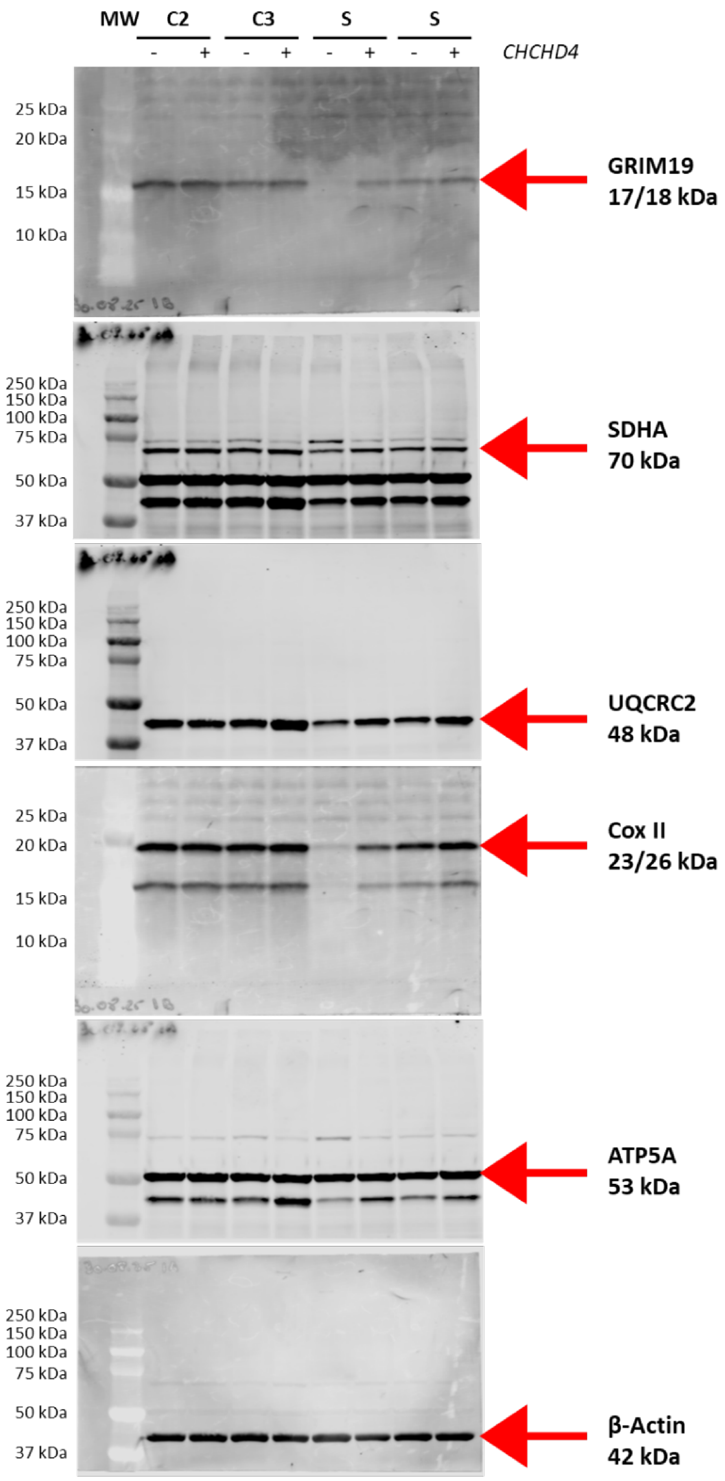

Biochemical investigation of fibroblasts. Full image of western blot analysis of OXPHOS subunits and β-actin in fibroblasts of subject and two controls (C2, C3) transduced with or without wt *CHCHD4* cDNA. MW: molecular weight.

Figure S4

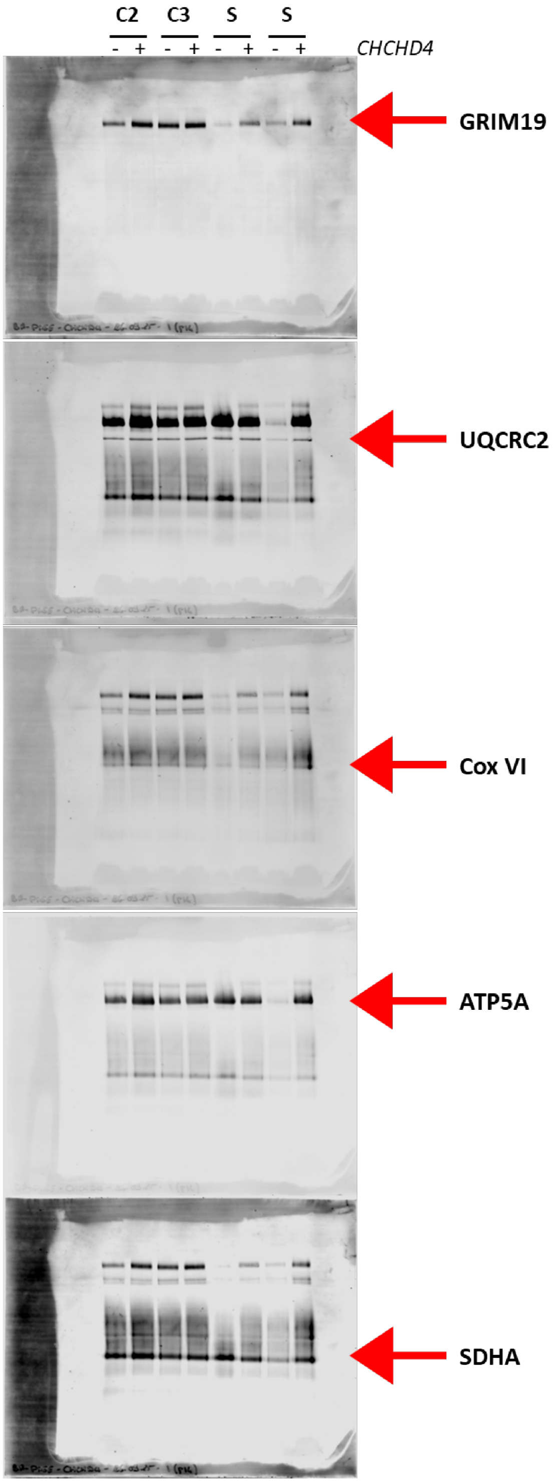

Biochemical investigation of fibroblasts. Full images of BN-PAGE on fibroblasts from subject (S) and two controls (C2, C3) transduced with or without wt *CHCHD4* cDNA.

HP:0001511	Intrauterine growth retardation
HP:0001943	Hypoglycemia
HP:0003128	Lactic acidosis
HP:0001410	Decreased liver function
HP:0001263	Global developmental delay
HP:0001298	Encephalopathy
HP:0012443	Abnormal brain morphology
HP:0025045	Abnormal brain lactate level by MRS

Table S1. Summary of clinical features of the subject Phenotypic findings of the subject mapped to HPO terms.

Table S2 is supplied as an Excel file

Table S2. Proteomic quantitative matrix and associated statistics combined with mitochondrial specific proteins from Mitocarta and over representation analysis of the down-regulated proteins from S_vs_S+CHCHD4 statistical analysis.

Supplemental Methods

Cell culture

Skin fibroblasts were grown in 5% CO2 at 37°C in Dulbecco's modified Eagle medium (DMEM, Gibco) supplemented with 4.5 g/L of D-glucose, 110 mg/L of sodium pyruvate, 10% fetal bovine serum (FBS, Gibco), and 100 U/mL of penicillin-streptomycin (Gibco).

Whole exome sequencing (WES)

Molecular genetic investigations were undertaken using EDTA-blood DNA samples. WES was performed on Subject as family-trio. WES data were interpreted according to American College of Medical Genetics (ACMG) guidelines1. The pathogenic variants were selected after filtering against known SNPs reported in dbSNP, 1000 Genomes, Exome Variant Server, in-house polymorphisms, and intergenic variants as previously described2.

Whole-cell protein extracts

Whole-cell protein extractions were carried out by suspending the cell pellets in RIPA buffer (ThermoFischer Scientific) supplemented with 1x Complete™ Protease Inhibitor Cocktail (Roche). Following an incubation on ice for 30 minutes the suspensions were centrifuged for 30 minutes at 16000 g in a cooled centrifuge. The supernatants were collected and stored at -20°C.

SDS-PAGE and immunoblotting

The protein concentration of the cell lysates was determined using BCA (ThermoFischer Scientific). 15 40 µg of whole-cell extracts, supplemented with Laemli buffer (Bio-Rad, Marnes-la-Coquette, France) and 100mM DTT (Merck), were fractionated through precast AnyKD Criterion TGX polyacrylamide gels (Bio-Rad) and blotted onto PVDF membranes (Bio-Rad). For analysis of the abundance of CHCHD4, samples were fractionated through AnyKD gels and immunoblotting was carried out using the following antisera: anti-CHCHD4 (HPA034688, Sigma Aldrich) and GAPDH (10494-1-AP, ProteinTech). OXPHOS proteins were fractionated through AnyKD gels and detected using the following antisera: anti-Grim19 (10986-1-AP, ProteinTech) for Complex I, anti-SDHA (ab14715, AbCam) for Complex II, 4 anti-UQCRC2 (ab14745, AbCam) for Complex III, anti-Cox II (55070-1-AP, ProteinTech) for Complex IV, anti-ATP5A (ab14748, AbCam) for Complex V and -anti-β-Actine conjugated with Alexa Fluor® 790 (ab184576, AbCam). IR Dye-conjugated (680RD or 800CW), secondary mouse or rabbit antisera were from Li-Cor Biosciences (Bad Homburg, Germany). Immunoblots were visualized with the Odyssey CLX infrared scanner (LI-COR Biosciences) and quantified using LI-COR's Image Studio.

BN-PAGE

For blue-native gel electrophoresis (BN-PAGE), mitoplasts were extracted from ~1x106 fibroblasts by incubation in 200 µl PBS containing 2 mg/ml Digitonin (Calbiochem) for 10 min on ice followed by dilution with 1 ml of PBS and centrifugation at 16000 x g for 10 min in a chilled centrifuge. The

mitoplast-containing pellets were washed once with PBS and resuspended in 60 µl ACBT buffer (1.5 M aminocaproic acid and 75 mM Bis-Tris (Sigma-Aldrich)) supplemented with 2% n-Dodecyl β-D maltoside (Sigma-Aldrich). After incubation on ice for 10 min, samples were centrifuged in a prechilled centrifuge at 16000 g for 30 min. Protein concentration in the extracts was determined using Bradford reagent (Sigma-Aldrich) and 10-15 µg of the extract were supplemented with Native PAGE sample buffer and Coomassie Brilliant Blue G-250 (ThermoFischer Scientific) prior to their fractionation through 4-16% NativePAGE Bis-Tris gel (ThermoFischer Scientific). Separated complexes were blotted onto a Immobilon-FL PVDF membrane (Millipore). OXPHOS complexes were detected using antibodies reactive against individual proteins from the five complexes: anti-Grim19 (10986-1-AP, ProteinTech) for complex I, anti-SDHA (ab14715, Abcam) for complex II, anti-UQCRC2 (ab147495, Abcam) for complex III, anti-COXIV (11242-1-AP, Proteintech) for complex IV and anti-ATP5A (Abcam, ab14748, Abcam) for complex V.

CHCHD4 overexpression

Fibroblasts grown in regular culture medium were transduced with human CHCHD4 cDNA cloned into the pD2109-CMV lentiviral vector (Atum), as previously described 3. Puromycin (Gibco) was added 72 hours after transduction at a final concentration of 5 µg/ml and selection was performed for 2 weeks during which media was changed every two days.

Proteomics analysis

5 S-TrapTM plate (Protifi, Hutington, USA) digestion was performed on 30 µg of cell lysates according to manufacturer's instructions. Briefly, samples were supplemented with 20% SDS to a final concentration of 5%, reduced with 20mM TCEP (Tris(2-carboxyethyl) phosphine hydrochloride) and alkylated with 50 mM chloracetamide (CAA) for 5 min at 95oC. Aqueous phosphoric acid was then added to a final concentration of 2.5% followed by the addition of S-Trap binding buffer (90% aqueous methanol, 100 mM TEAB, pH7.1). Mixtures were then loaded on S-Trap plate. Five washes were performed for thorough SDS elimination. Samples were digested with 1.5 µg of trypsin (Promega) at 47°C for 2 h. After elution, peptides were vacuum dried and resuspended in 2% ACN, 0.1% formic acid in HPLC-grade water prior to MS analysis. Peptides were resuspended in 30 µL of 2% ACN, 0.1% formic acid in HPLC-grade water and 300 ng were injected on an Evosep One system coupled to a timsTOF HT (Bruker Daltonics, Germany) mass spectrometer. The Evosep One system operated with the Whisper Zoom 40 Samples Per Day method using a 15 cm C18 Aurora Elite column (AUR3-15075C18-CSI, IonOpticks). The mobile phases comprised 0.1% FA as solution A and 0.1% FA/99.9% ACN as solution B. Mass-spectrometric data were acquired using the parallel accumulation serial fragmentation (PASEF) acquisition method in DIA (Data independent Analysis) mode with a 21-windows method using 25 Da windows covering the mobility ranges over a 475-1000 m/z range. The range of ion mobilities values from 0.85 to 1.27 V s/cm2 (1/k0). The total cycle time was set to 0.95 s. Data analysis was

performed using DIA-NN software (version 1.8.2). A search against the human UniProtKB/Swiss-Prot Homo sapiens database (downloaded the 12th of February, 2025, 20417 entries) was performed using library free workflow. For this purpose, "FASTA digest for library free search/library generation" and "Deep learning spectra, RTs and IMs prediction" options were checked for precursor ion generation. A maximum of 1 trypsin missed cleavages was allowed and the maximum variable modification was set to 2. Carbamidomethylation (Cys) was set as the fixed modification, whereas protein N-terminal methionine excision, methionine oxidation and N-terminal acetylation 6 were set as variable modifications. The peptide length range was set to 7–30 amino acids, precursor charge range 2–4, precursor m/z range 300–1300, and fragment ion m/z range 300–1300. To search the parent mass and fragment ions, accuracy was set to 10 ppm manually. The false discovery rates (FDRs) at the protein and peptide level were set to 1%. Match between runs was allowed. For the quantification strategy, Robust LC (high precision) was used as advised in the software documentation, whereas default settings were kept for the other algorithm parameters. Statistical and bioinformatic analysis were performed with MassDynamics 2.0 software available at https://massdynamics.com. All R figures was created using R (version 4.4) and RStudio (version 2025.09). The majority of the figures were created using ggplot2 (v3.5.2) embedded in the tidyverse (v2.0). In particular for Principal Component Analysis (PCA) and heatmap representation, the PCAtools (v2.18.0) and ComplexHeatmap R (v2.22) package were used, respectively. Over Representation Analysis using Cellular Component Gene Ontology (GO CC) and subsequent representation of the results was performed using ClusterProfiler (v4.14.6), enrichplot (v1.27.4) and org.Hs.eg.db (v3.20.0) R packages, respectively. Data have been deposited to the ProteomeXchange Consortium via the PRIDE4 partner repository with the dataset identifier PXD069027.

Supplemental References

1. Richards, S., Aziz, N., Bale, S., Bick, D., Das, S., Gastier-Foster, J., Grody, W.W., Hegde, M., Lyon, E., Spector, E., et al. (2015). Standards and guidelines for the interpretation of sequence variants: a joint consensus recommendation of the American College of Medical Genetics and Genomics and the Association for Molecular Pathology. Genet Med 17, 405-424. 2. Thompson, K., Bianchi, L., Rastelli, F., Piron-Prunier, F., Ayciriex, S., Besmond, C., Hubert, L., Barth, M., Barbosa, I.A., Deshpande, C., et al. (2022). Biallelic variants in TAMM41 are associated with low muscle cardiolipin levels, leading to neonatal mitochondrial disease. HGG Adv 3, 100097.

3. Gardeitchik, T., Mohamed, M., Ruzzenente, B., Karall, D., Guerrero-Castillo, S., Dalloyaux, D., van den Brand, M., van Kraaij, S., van Asbeck, E., Assouline, Z., et al. (2018). Bi-allelic Mutations in the Mitochondrial Ribosomal Protein MRPS2 Cause Sensorineural Hearing Loss, Hypoglycemia, and Multiple OXPHOS Complex Deficiencies. Am J Hum Genet 102, 685-695.

4. Perez-Riverol, Y., Bandla, C., Kundu, D.J., Kamatchinathan, S., Bai, J., Hewapathirana, S., John, N.S., Prakash, A., Walzer, M., Wang, S., et al. (2025). The PRIDE database at 20 years: 2025 update. Nucleic Acids Res 53, D543-D553.
