# Supplementary material for: Biallelic variants in CHCHD4 are associated with combined OXPHOS defect leading to mitochondrial disease
Source: HGG Adv. 2026 Apr 14;7(3):100615. doi: 10.1016/j.xhgg.2026.100615 (PMC13147372; doi:10.1016/j.xhgg.2026.100615)
Supplement: Document S2. Article plus supplemental information [file mmc3.pdf]

HGG Advances

BRIEF COMMUNICATION

Biallelic variants in *CHCHD4* are associated with combined OXPHOS defect leading to mitochondrial disease

Matthieu Mantecon,[1] Cerina Chhuon,[2] Kevin Roger,[2] Ida Chiara Guerrera,[2] Christine Bole,[3] Patrick Nitschke,[4] Claire-Marie Dufeu-Bérat,[5] Margaret Ashcroft,[6] Robert W. Taylor,[7,8] Nathalie Boddaert,[9] and Agnès Rötig[1,10,*]

Summary

Mitochondrial disorders show remarkable clinical and genetic heterogeneity and result from variants in either mitochondrion- or nucleus-encoded genes. CHCHD4 is a component of the mitochondrial import and assembly pathway that imports small cysteine-containing substrates. We report a pediatric patient with biallelic *CHCHD4* variants who presented with severe neurological regression and early death. Western blot analysis showed decreased levels of CHCHD4 and diminished assembly of complexes I and IV in his fibroblasts. To demonstrate that *CHCHD4* variants were responsible for the observed biochemical phenotype, we overexpressed wild-type *CHCHD4* in control and subject fibroblasts, restoring levels of complex I and IV proteins and the associated assembly defects. Proteomic studies pointed to electron transport and complex I biogenesis as the main dysregulated pathways and showed a severe loss of several complex I and IV proteins and/or assembly factors rescued by overexpression of wild-type *CHCHD4*. CHCHD4 has numerous targets and interacting factors and is involved in the export of iron-sulfur clusters synthesized inside mitochondria. Surprisingly, few of these interacting factors or non-mitochondrial functions were impacted by the observed CHCHD4 defect. In conclusion, our work establishes CHCHD4 deficiency as a cause of dysregulated mitochondrial protein import resulting in a severe neurological condition.

Introduction

Most mitochondrial proteins are encoded by nuclear genes and subsequently imported into the organelle through the outer-membrane translocase (TOM), the sorting and assembly machinery (SAM), and the inner-membrane translocase (TIM).[1] A subgroup of small cysteine-containing mitochondrial proteins carrying $(CX_9C)_2$ motifs is imported by the evolutionary conserved mitochondrial import and assembly (MIA) pathway, located in the intermembrane space (IMS). The MIA pathway, which catalyzes the oxidative folding of incoming mitochondrial proteins through a disulfide relay system (DRS), is composed of CHCHD4 and growth factor ERV1-like (GFER).[2] CHCHD4 imports small cysteine-containing substrates, such as TIM proteins (TIMM8A and TIM13), complex I subunits (NDUFA8, NDUFB7, NDUFB10, NDUFS5, and NDUFS8), complex IV subunits and assembly factors (COX6B1, COX17, COX19, COA4, COA5, and COA6), or the regulator of mitochondrial Ca^{2+} uniporter, expanding the diversity of CHCHD4 substrates.[3] The CHCHD-containing proteins are $(CX_9C)_2$ motif-containing proteins involved in several mitochondrial functions. CHCHD1 is a subunit of the mitoribosome, CHCHD3 and CHCHD6 are components of the mitochondrial contact site and crista-organizing system (MICOS), CHCHD10 is involved in MICOS stability, and CHCHD8 is an assembly factor of complex IV.[4] CHCHD4 is the only known CHCHD-containing protein involved in protein import into the IMS. It also interacts with the apoptosis-inducing factor AIFM1, which acts as an effector of CHCHD4 activity.[5] CHCHD4 contains an iron-sulfur cluster (ISC) and is also a component of the mitochondrial ISC export machinery, modulating cellular iron homeostasis.[6] Finally, CHCHD4 can regulate mitochondrial DNA (mtDNA) release into the cytosol, triggering the cGAS-STING-nuclear factor κB (NF-κB) pathway.[7]

Pathogenic variants in genes encoding CHCHD proteins such as *CHCHD2* (MIM: 616244; coiled-coil-helix-coiled-coil-helix domain-containing protein 2) and *CHCHD10* (MIM: 615903; coiled-coil-helix-coiled-coil-helix domain-containing protein 10) have been reported in Parkinson disease[8] and in frontotemporal dementia and/or amyotrophic lateral sclerosis-2,[9] respectively. Moreover, *GFER* (MIM: 600924) variants result in mitochondrial myopathy

[1]Université Paris Cité, Institut Imagine, Genetics of Mitochondrial Diseases, INSERM UMR, 1163 Paris, France; [2]Proteomics Platform Necker, Université Paris Cité-Structure Fédérative de Recherche Necker, INSERM US24/CNRS UAR3633, 75015 Paris, France; [3]Université Paris Cité, Institut Imagine, Genomics Platform, 75015 Paris, France; [4]Université Paris Cité, Institut Imagine, Bioinformatic Platform, 75015 Paris, France; [5]Departments of Pediatrics, Hôpital Necker-Enfants Malades, AP-HP, Université Paris Cité, 75015 Paris, France; [6]University of Cambridge, Department of Medicine, Cambridge Biomedical Campus, Hills Road, Cambridge CB2 0QQ, UK; [7]Mitochondrial Research Group, Clinical and Translational Research Institute, Faculty of Medical Sciences, Newcastle University, Newcastle upon Tyne NE2 4HH, UK; [8]NHS Highly Specialised Service for Rare Mitochondrial Disorders, Newcastle upon Tyne Hospitals NHS Foundation Trust, Newcastle upon Tyne NE1 4LP, UK; [9]Pediatric Radiology Department, AP-HP, Hôpital Necker Enfants Malades, Université Paris Cité and Institut Imagine INSERM U1163, 75015 Paris, France
[10]Lead contact
*Correspondence: agnes.rotig@inserm.fr
https://doi.org/10.1016/j.xhgg.2026.100615.
Crown Copyright © 2026 Published by Elsevier Inc. on behalf of American Society of Human Genetics.
This is an open access article under the CC BY license (http://creativecommons.org/licenses/by/4.0/).

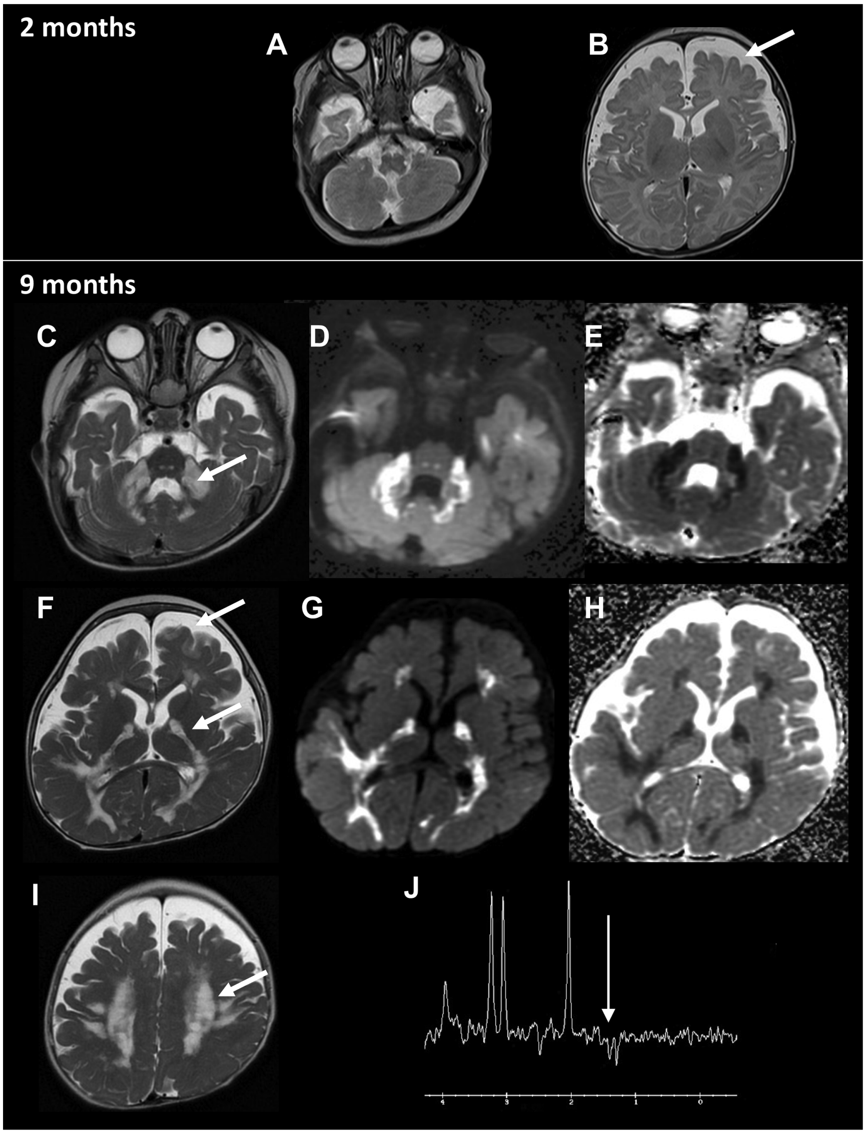

Figure 1. Brain MRI of the subject at 2 and 9 months of age

(A and B) Brain MRI axial slices in T2. The arrow indicates the enlargement of the periventricular spaces in the supratentorial region.

(C–I) Illustration of brain images at 9 months of age. Shown are brain MRI axial slices on T2 (C, F, and I) and diffusion weighted images (D and G) and apparent diffusion coefficients (E and H). The arrows indicate marked hypersignal in middle cerebellar peduncles (C), semioval centers (I), pyramidal tracts (F), and marked enlargement of the periventricular spaces in the supratentorial region (F).

(J) MR spectroscopy showed a lactate peak (arrow).

with cataracts (MIM: 613076),[10] and *AIFM1* (MIM: 300169) variants cause mitochondrial disease manifesting as combined oxidative phosphorylation (OXPHOS) deficiency (MIM: 300816; combined oxidative phosphorylation deficiency 6 [COXPD6]).[11] To date, there are no reports of pathogenic *CHCHD4* (MIM: 611077) variants. Here, we report a subject carrying bi-allelic *CHCHD4* variants who presented with severe lactic acidosis and psychomotor and neurological regression. Using subject-derived fibroblasts, we demonstrate impaired OXPHOS function that can be restored by expression of wild-type (WT) *CHCHD4* cDNA, while an unbiased proteomic analysis allows us to decipher the mitochondrial and non-mitochondrial consequences of CHCHD4 deficiency.

Subjects and methods

The subject was the first child of non-consanguineous healthy parents, with no family history. Pregnancy was uneventful except for gestational hypertension diagnosed at 37+2 weeks. The infant was born at 37+3 weeks following induction for abnormal fetal heart rate tracing. Apgar scores were 9/10/10. Intrauterine growth restriction was noted (birth weight: 1,860 g, <third percentile; head circumference: 31.5 cm, third percentile; Table S1). Birth length was not documented. At 9 h of life, the infant presented with persistent hypoglycemia refractory to oral glucose supplementation, requiring continuous intravenous glucose infusion. Metabolic workup revealed severe lactic acidosis (pH 7.00, lactate 26 mmol/L), hepatocellular cytolysis (AST, 176 U/L; ALT, 42 U/L), cholestasis, and moderate hyperammonemia (135 μmol/L), consistent with acute liver dysfunction. Further investigations showed massive lactic aciduria. Rapid normalization of hepatic function and acid-base balance occurred within the first days of life. However, recurrent short-fasting hypoglycemia persisted, requiring enriched feeding regimens and nocturnal enteral nutrition. Chronic hyperlactatemia (2–6.5 mmol/L) persisted, with an elevated lactate/pyruvate ratio (blood lactate/pyruvate ratio [L/P] = 33.5). From 6 months of age, glycemic control improved progressively. However, a delay in developmental milestones was noted, with increased peripheral hypertonia. At 9 months, the patient developed psychomotor development regression with loss of previously acquired motor skills and impaired social interaction. Neurological deterioration progressed rapidly, with episodes of inconsolable crying, marked hypertonia, and major discomfort. He had worsened hyperlactatemia (7.5 mmol/L), elevated cerebrospinal fluid lactate (4.5 mmol/L), and increased L/P (cerebrospinal fluid [CSF] L/P = 27.7). The neurological phenotype evolved toward a severe encephalopathic state with recurrent dystonic episodes and major discomfort. The patient died at 11 months of age.

A first brain MRI performed at 2 months of age showed enlargement of the periventricular spaces in the supratentorial region (Figure 1). At 9 months of age, brain MRI detected hypersignal in the white matter of the semioval centers, the pyramidal tracts, the brain stem, and the middle cerebellar peduncles. Diffusion

A

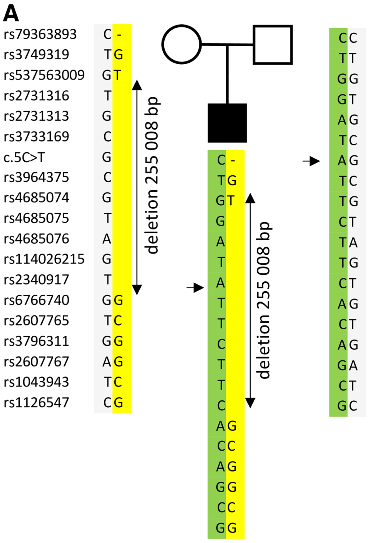

B

nucleotide (NM_001098502.1)	c.5C>T
nucleotide (genomic)	Chr3(GRCh38):g.14124672G>A
amino acid	p.Ser2Phe
CADD (v1.6)	Raw score: 0.595131
REVEL (v2021-05-03)	Score: 0.003
Align GVGD	C0
SIFT	0.04 (deleterious)
MutationTaster	disease causing (p-value: 1)
Polyphen	0.832
AlphaMissense Pathogenicity	0.956 (likely pathogenic)

C

```
               Ser2            Asp19
human      --MSYCRQEGKDRIIFVTKEDHETPSSAELVADD
mouse      --MSYCRQEGKDRIIFVTKEDHETPSSAELVADD
xenopus    --MSYCRQEGKDKIIFVTKEDHETPSSAELIADD
tetraodon  --MSYCKQEGKDRIIFVTKEDHDTPSNAELIAED
zebrafish  --MSYCKQEGKDCIIFVTKEDHEAPSNAELVEDD
droso      MSFSYCKEYGKDKVIFVTKEDHATPSTIELPP--
```

D

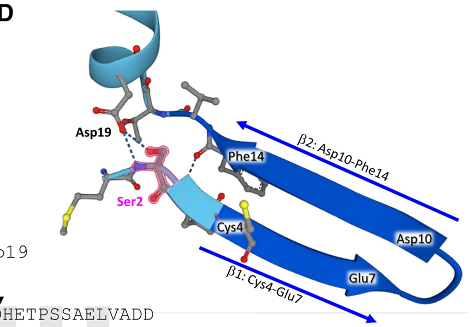

Figure 2. CHCHD4 variants

(A) Pedigree of the subject's family and haplotypes at the *CHCHD4* locus. The mutant paternal and maternal alleles are indicated in green and yellow, respectively. The arrow indicates the c.5C>T variant. The maximum size of the deletion is 255,753 bp (between heterozygous rs537563009 and rs6766740 SNPs), and the minimum size is 22,215 bp (between homozygous rs2731316 and rs2340917 SNPs).

(B) Evidence of pathogenicity associated with the c.5C>T *CHCHD4* variant.

(C) Multisequence alignment of CHCHD4 proteins.

(D) Three-dimensional representation of the human CHCHD4 structure predicted by AlphaFold, showing the location and the prediction of hydrogen-bonding (H-bond, dotted lines) of Ser2 (pink). The two arrows indicate the β strands of the N-terminal part of the protein. The colors of the 3D structure correspond to the confidence scores of the different parts of the protein: dark blue, very high (pLDDT > 90); light blue, confident (90 > pLDDT >70); yellow, low (70 > pLDDT >50); and orange, very low (pLDDT <50).

weighted imaging showed apparent diffusion coefficient restriction in the pathological areas. Spectroscopy revealed a lactate peak. No abnormality in the basal ganglia could be observed.

Informed consent was obtained for the subject in accordance with the Declaration of Helsinki protocols and approved by local institutional review boards in Paris.

Details of cell culture, whole-exome sequencing, protein extraction, SDS-PAGE and immunoblotting, BN-PAGE, CHCHD4 overexpression, and proteomics analysis are presented as supplemental information.

Results

Molecular genetic investigations were undertaken using EDTA-blood DNA samples. mtDNA sequencing did not detect any pathological variant. WES was performed on the subject as a family trio. A first WES analysis in the trio did not allow identification of any gene with biallelic heterozygous variations predicted to be deleterious. Nevertheless, analysis of WES data of the subject alone resulted in the identification of an apparently homozygous variant in *CHCHD4* (NM_001098502.1, *CHCHD4.1*), also known as MIA40. This c.5C>T variation (p.Ser2Phe) was inherited from the father, but the mother was apparently homozygous for the WT allele, which prompted us to hypothesize that instead she carried a deletion at the *CHCHD4* locus. Indeed, analysis of the WES data at *CHCHD4* locus showed loss of heterozygosity of several successive SNPs in the mother and her son, confirming the presence of a large deletion of 255 kb maximum, encompassing the complete CHCHD4 gene and exons 1–7 of the adjacent *TMEM43* gene (Figure 2A). The CHCHD4 Ser2Phe change replaces a hydrophilic with a hydrophobic amino acid; this change affects a highly evolutionary conserved amino acid and is predicted to be deleterious (Figures 2B and 2C). TMEM43 is a nuclear envelope protein involved in the organization of protein complexes at the inner nuclear membrane.[12] Heterozygous *TMEM43* missense or nonsense variations result in adult-onset autosomal-dominant arrhythmogenic right ventricular dysplasia,[13] Emery-Dreifuss muscular dystrophy,[14] or auditory neuropathy,[15] but no large deletion has been previously reported. Considering that the mother is healthy, we hypothesize that the *TMEM43* deletion has no clinical consequences.

To visualize the location of mutant amino acid, we used the AlphaFold machine learning algorithm that predicts protein structures and that has high confidence in the location of the identified missense variant.[16] Ser2 is located just before the β1 strand (Cys4-Glu7), forming a β hairpin with the β2 strand.[17] Ser2 is predicted by AlphaFold to be involved in two hydrogen-bonding (H bonds) with Asp19. Both Ser2 and Asp19 are highly conserved residues among species, suggesting they are important residues for CHCHD4 protein function and/or structure (Figure 2D).

SDS-PAGE and western blot analysis revealed a decreased steady-state level of CHCHD4 protein in cultured skin

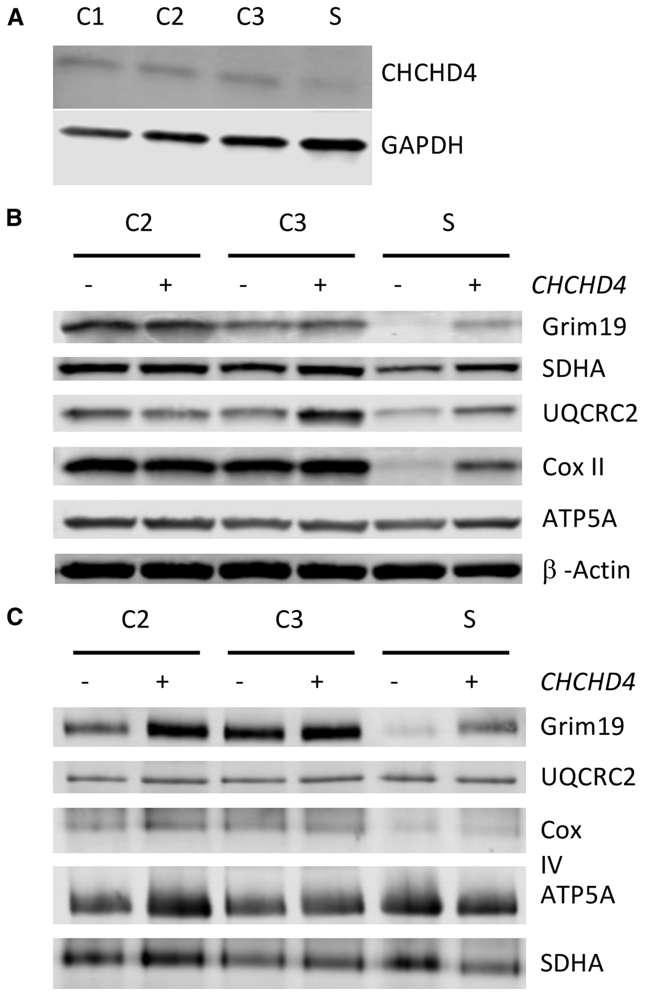

Figure 3. Biochemical investigation of fibroblasts
(A) Western blot analysis of CHCHD4 performed on total protein of fibroblasts of the subject (S) compared to 3 controls (C1, C2, and C3). GAPDH was used as a loading control.
(B) Western blot analysis of OXPHOS subunits in fibroblasts of the subject and two controls (C2 and C3) transduced with or without WT *CHCHD4* cDNA. β-actin was used as a loading control.
(C) BN-PAGE on fibroblasts from S and 2 controls transduced with or without WT *CHCHD4* cDNA.

fibroblasts of the subject compared to controls (Figures 3A and S2), suggesting that the *CHCHD4* variants affect the stability of the protein. CHCHD4 mediates the import of several mitochondrial proteins, including complex I and complex IV subunits and/or assembly factors. Consistent with this, we could observe a severe decrease of various OXPHOS subunits, Grim19, and UQCRC2 in fibroblasts with a relative sparing of ATP5A (Figures 3B and S3). Moreover, the mitochondrially encoded COXII subunit was also decreased, suggesting a destabilization of at least OXPHOS complex IV. Finally, to assess the assembly status and function of the OXPHOS system, we carried out BN-PAGE analyses, which revealed an obvious complex I and IV assembly defect (Figures 3C and S4).

To demonstrate that variations in the *CHCHD4* gene were responsible for the observed molecular phenotype of subject-derived fibroblasts and therefore are likely disease causing in nature, we undertook functional complementation analyses in control and subject-derived fibroblasts. Fibroblasts were transduced with lentiviral particles expressing WT *CHCHD4* using pD2109 (Atum) for 12 h and then incubated for an additional 40 days until the cells were harvested. Stable transduction with lentiviral particles expressing WT *CHCHD4* cDNA (transcript variant 1) restored the steady-state levels of GRIM19, UQCRC2, and COXII proteins as well as a complex I and IV assembly defect (Figures 3B and 3C).

To assess the effect of the identified *CHCHD4* variants on various cellular functions, we performed proteomics analysis in subject and control fibroblasts transduced with or without WT *CHCHD4* cDNA. The proteomics analysis allowed the quantification of 8211 proteins, 603 of which were significantly downregulated in the fibroblasts of the subject compared to the control group (t test, false discovery rate [FDR] = 0.05). Overall, mitochondrial proteins were found to be mostly downregulated (17% of the downregulated proteins were mitochondrial proteins against 3% of the upregulated proteins) (Figure 4A; Table S2). CHCHD4 was significantly decreased in subject fibroblasts (Figure 4A) and transduction with WT *CHCHD4* cDNA significantly increased its level in subject-derived fibroblasts (Figure S1C). As expected, the TMEM43 protein level was reduced in subject-derived fibroblasts compared to the control and not modified by overexpression of WT *CHCHD4* cDNA (Figure S1C).

Reactome pathway analysis identified respiratory electron transport and complex I biogenesis as the main dysregulated pathways within our dataset (FDR = 1.4e−14; Figures S1A and S1B; Table S2). We then systematically looked at OXPHOS structure proteins and assembly factors and detected decreased levels of a large part of nucleus-encoded complex I subunits (Figures 4B and 4C; Table S2). Among them, only NDUFA8, NDUFB8, NDUFB10, and NDUFS5 are known CHCHD4 targets,[2] suggesting a secondary defect of other subunits related to complex I assembly defect. Most complex IV subunits were also significantly decreased (Figures 4B and 4D; Table S2). Two complex IV assembly factors, COA7 and COA6, were also reduced (FC −3.6 and −2.76, respectively) as well as SCO2, COX19, COX11, and COX17 to a lesser extent (FC −2.08 to −1.07; Table S2). Accordingly, COX6B1 complex IV subunit and the COA6, COA7, SCO2, and COX17 assembly factors are CHCHD4 targets.[2] No significant modification of nucleus-encoded subunits or assembly factors of complexes II, III, and V was observed (data not shown). Interestingly, several complex I and IV subunits encoded by mtDNA were also significantly decreased, but they do not require mitochondrial import, as they are synthesized within mitochondria (Figure 4E; Table S2). This decrease should be related to the decreased stability of complexes I and IV rather to abnormal mitochondrial protein synthesis, as factors

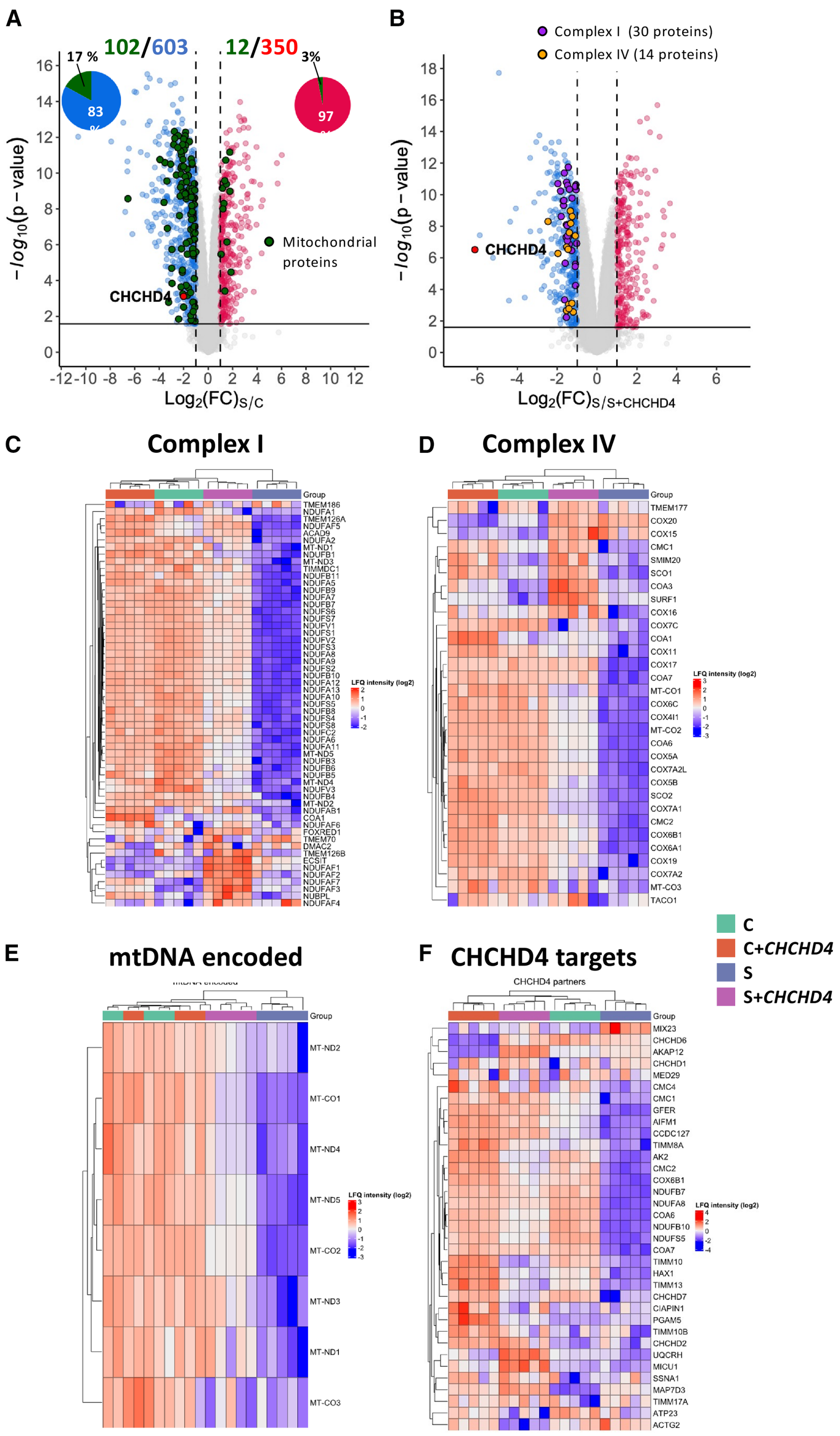

Figure 4. Proteomics analysis of subject and control fibroblasts transduced or not by WT *CHCHD4* cDNA

(A) Volcano plot representing the statistical comparison of the protein label-free quantification (LFQ) intensities of subject fibroblasts vs. control fibroblasts (FC = 2, FDR = 0.05). Proportions of up- and downregulated mitochondrial proteins are reported. Mitochondrial proteins (green) were retrieved from Mitocarta and literature data. CHCHD4 is shown in red.

(B) Volcano plot representing the statistical comparison of the protein LFQ intensities of subject fibroblasts vs. subject fibroblasts transduced with WT *CHCHD4* cDNA (FC = 2, FDR = 0.05). Complex I and IV proteins are reported.

(C–F) Heatmap representation depicting the dysregulated complex I (C) and complex IV (D) subunits and/or assembly factors, mtDNA-encoded proteins (E), and CHCHD4 targets (F) in different samples.

involved in mitochondrial translation were mostly not changed in the subject's fibroblasts (Table S2), whereas downregulation of CHCHD4 has been previously shown to decrease several mitoribosome proteins.[18] Interestingly, overexpression of WT *CHCHD4* cDNA in subject's fibroblasts increased the level of almost all of these proteins (Figures 4 and S1C; Table S2), demonstrating that *CHCHD4* variants are the cause of the OXPHOS defect and that improvement of protein import significantly rescues OXPHOS function.

CHCHD4 forms a stable complex with AIFM1 through a direct interaction with its N-terminal part[19] for mitochondrial protein import, but only a minor decrease of AIFM1 was observed in the subject's fibroblasts (Figure S1C; Table S2). This is consistent with previous reports showing that knockdown of CHCHD4 does not affect the AIF amount.[19] CHCHD4 also interacts with GFER protein, essential for protein folding and import of a subset of mitochondrial proteins as part of the DRS. Notably, CHCHD4 downregulation has been shown to decrease the mitochondrial import of GFER.[20] Accordingly, the subject's fibroblasts displayed a medium decrease of GFER, and overexpression of WT *CHCHD4* cDNA increased it in the subject's fibroblasts (Figure S1C; Table S2).

CHCHD4 has a central role in mitochondrial protein import and has several targets and interacting factors. CHCHD-containing proteins are imported with the help of the CHCHD4 import machinery,[2] but no major modification of these proteins was observed in subject-derived cells (Figure 4F; Table S2). Similarly, very few components of translocase of outer and inner mitochondrial membrane machinery were modified despite several proteins

of this machinery being substrates for CHCHD4[21] (Figure 4F; Table S2). Finally, the additional CHCHD4 partner proteins[22,23] were not significantly modified in the subject's fibroblasts except those involved in complexes I and IV (Figure 4F; Table S2).

CHCHD4 is not only involved in mitochondrial protein import but also in the export of ISCs synthesized inside the mitochondria[6] that are then incorporated in cytosolic or nuclear proteins involved in essential cellular pathways such as iron homeostasis (IRP2 and FBXL5), nuclear DNA polymerases, helicases or exonucleases, or interferon response (RSAD2).[24–26] Accordingly, CHCHD4 downregulation in HEK293 cells lowered the activity and stability of several cytosolic ISC-containing proteins.[6] Among all known non-mitochondrial ISC-containing proteins, only one, the AOX1 aldehyde oxidase, a cytosolic drug-metabolizing enzyme,[27] was significantly decreased in the subject's fibroblasts (FC −3.26) and increased by overexpression of WT *CHCHD4* cDNA (Figure S1C).

Finally, none of the proteins and enzymes involved in mtDNA maintenance, mtRNA metabolism, nucleotide metabolism, the tricarboxylic acid cycle, mitochondrial lipid metabolism, fatty acid oxidation, CoQ_{10} synthesis, mitochondrial dynamics and surveillance, amino acid metabolism, or mitochondrial transport were downregulated in CHCHD4-deficient fibroblasts (data not shown), suggesting that these proteins do not depend on CHCHD4 for protein import. In keeping with this, neither the regulator of the mitochondrial Ca^{2+} uniporter MICU1, a well-known target of CHCHD4,[23] nor other proteins involved in mitochondrial calcium metabolism were changed in the subject's fibroblasts (data not shown). Furthermore, proteomics analysis did not detect other non-mitochondrial deregulated pathways, suggesting that *CHCHD4* variants primarily affect OXPHOS proteins.

Discussion

In conclusion, this work shows that a CHCHD4 (MIA40) defect in humans is associated with a severe mitochondrial disease, resulting mainly from an OXPHOS function and assembly defect. Nevertheless, this defect does not alter other well-known functions depending on CHCHD4, such as Ca^{2+} signaling, or cytosolic ISC-containing proteins, suggesting that the residual amount of CHCHD4 is sufficient to maintain these functions. This confirms a central role of CHCHD4 in OXPHOS protein import, but why other functions are not affected is intriguing. Moreover, these results add to rare causes of protein import defects associated with pathogenic variants in *AIFM1*, *GFER*, *TIMM50* (MIM: 607381; translocase of inner mitochondrial membrane 50), *TIMM8B* (MIM: 606659), *TIMM22* (MIM: 607251), *TOMM7* (MIM: 607980; translocase of outer mitochondrial membrane 7), *MAGMAS/PAM16* (MIM: 614336; presequence translocase-associated motor 16), and *DNAJC19* (MIM: 608977, DNAJ/HSP40 homolog, subfamily C, member 19)[28] and emphasizes the wide clinical heterogeneity associated with this cause of genetic diseases.

Limitations of the study

The coexistence of a complete *CHCHD4* deletion and the p.Ser2Phe variation leads to a significant decrease in CHCHD4 protein, and it is not possible to determine whether the p.Ser2Phe variation leads to a total loss of protein function or whether some residual activity remains.

We present here a single case that does not allow us to determine the natural history of CHCHD4, and identification of additional individuals with *CHCHD4* variants will allow us to better define the clinical and biochemical consequences of these very rare variations.

Data and code availability

The genome sequencing data supporting the current study have not been deposited in a public repository because consent was not obtained for this.

The published article includes all other data generated or analyzed during this study, or it is readily available from the authors.

Proteomic data submission

The mass spectrometry proteomics data have been deposited into the ProteomeXchange Consortium via the PRIDE partner repository with the dataset identifier PXD069027.

ClinVar submission

The accession number for the c.5C>T (p.Ser2Phe) variant is VCV004795219.1. The accession number for the CHCHD4 deletion is SCV007338468.

Acknowledgments

This study was financially supported by the Agence Nationale de la Recherche through the Investissements d'Avenir program ANR-10-IAHU-01 (to M.M., C.B., P.N., and A.R.) and the E-Rare project GENOMIT (01GM1207 to A.R. and M.M.). We acknowledge the use of bioresources of the Necker Imagine DNA Biobank (BB-033-00065). R.W.T. is funded by the Wellcome Centre for Mitochondrial Research (203105/Z/16/Z), the Mitochondrial Disease Patient Cohort (UK) (G0800674), the Medical Research Council (MR/W019027/1), the Lily Foundation, the Pathological Society, the UK NIHR Biomedical Research Centre for Ageing and Age-related disease award to the Newcastle upon Tyne Foundation Hospitals NHS Trust, LifeArc, and the UK NHS Highly Specialised Service for Rare Mitochondrial Disorders of Adults and Children. M.A. thanks Medical Research Council (MR/K002201/2).

Declaration of interests

The authors declare no competing interests.

Supplemental information

Supplemental information can be found online at https://doi.org/10.1016/j.xhgg.2026.100615.

Web resources

Alphafold Protein Structure Database, https://alphafold.ebi.ac.uk/

MassDynamics 2.0 software, https://massdynamics.com

OMIM, http://www.omim.org/

Received: October 10, 2025
Accepted: April 10, 2026

References

1. Pfanner, N., den Brave, F., and Becker, T. (2025). Mitochondrial protein import stress. Nat. Cell Biol. *27*, 188–201.
2. Modjtahedi, N., Tokatlidis, K., Dessen, P., and Kroemer, G. (2016). Mitochondrial Proteins Containing Coiled-Coil-Helix-Coiled-Coil-Helix (CHCH) Domains in Health and Disease. Trends Biochem. Sci. *41*, 245–260.
3. Al-Habib, H., and Ashcroft, M. (2021). CHCHD4 (MIA40) and the mitochondrial disulfide relay system. Biochem. Soc. Trans. *49*, 17–27.
4. Zhou, Z.D., Saw, W.T., and Tan, E.K. (2017). Mitochondrial CHCHD-Containing Proteins: Physiologic Functions and Link with Neurodegenerative Diseases. Mol. Neurobiol. *54*, 5534–5546.
5. Brosey, C.A., Shen, R., and Tainer, J.A. (2025). NADH-bound AIF activates the mitochondrial CHCHD4/MIA40 chaperone by a substrate-mimicry mechanism. EMBO J. *44*, 1220–1248.
6. Murari, A., Thiriveedi, V.R., Mohammad, F., Vengaldas, V., Gorla, M., Tammineni, P., Krishnamoorthy, T., and Sepuri, N.B.V. (2015). Human mitochondrial MIA40 (CHCHD4) is a component of the Fe-S cluster export machinery. Biochem. J. *471*, 231–241.
7. Ma, J., Wang, P.Y., Zhuang, J., Son, A.Y., Karius, A.K., Syed, A.M., Nishi, M., Wu, Z., Mori, M.P., Kim, Y.C., and Hwang, P.M. (2024). CHCHD4-TRIAP1 regulation of innate immune signaling mediates skeletal muscle adaptation to exercise. Cell Rep. *43*, 113626.
8. Funayama, M., Ohe, K., Amo, T., Furuya, N., Yamaguchi, J., Saiki, S., Li, Y., Ogaki, K., Ando, M., Yoshino, H., et al. (2015). CHCHD2 mutations in autosomal dominant late-onset Parkinson's disease: a genome-wide linkage and sequencing study. Lancet Neurol. *14*, 274–282.
9. Bannwarth, S., Ait-El-Mkadem, S., Chaussenot, A., Genin, E.C., Lacas-Gervais, S., Fragaki, K., Berg-Alonso, L., Kageyama, Y., Serre, V., Moore, D.G., et al. (2014). A mitochondrial origin for frontotemporal dementia and amyotrophic lateral sclerosis through CHCHD10 involvement. Brain *137*, 2329–2345.
10. Di Fonzo, A., Ronchi, D., Lodi, T., Fassone, E., Tigano, M., Lamperti, C., Corti, S., Bordoni, A., Fortunato, F., Nizzardo, M., et al. (2009). The mitochondrial disulfide relay system protein GFER is mutated in autosomal-recessive myopathy with cataract and combined respiratory-chain deficiency. Am. J. Hum. Genet. *84*, 594–604.
11. Ghezzi, D., Sevrioukova, I., Invernizzi, F., Lamperti, C., Mora, M., D'Adamo, P., Novara, F., Zuffardi, O., Uziel, G., and Zeviani, M. (2010). Severe X-linked mitochondrial encephalomyopathy associated with a mutation in apoptosis-inducing factor. Am. J. Hum. Genet. *86*, 639–649.
12. Bengtsson, L., and Otto, H. (2008). LUMA interacts with emerin and influences its distribution at the inner nuclear membrane. J. Cell Sci. *121*, 536–548.
13. Merner, N.D., Hodgkinson, K.A., Haywood, A.F.M., Connors, S., French, V.M., Drenckhahn, J.D., Kupprion, C., Ramadanova, K., Thierfelder, L., McKenna, W., et al. (2008). Arrhythmogenic right ventricular cardiomyopathy type 5 is a fully penetrant, lethal arrhythmic disorder caused by a missense mutation in the TMEM43 gene. Am. J. Hum. Genet. *82*, 809–821.
14. Liang, W.C., Mitsuhashi, H., Keduka, E., Nonaka, I., Noguchi, S., Nishino, I., and Hayashi, Y.K. (2011). TMEM43 mutations in Emery-Dreifuss muscular dystrophy-related myopathy. Ann. Neurol. *69*, 1005–1013.
15. Jang, M.W., Oh, D.Y., Yi, E., Liu, X., Ling, J., Kim, N., Sharma, K., Kim, T.Y., Lee, S., Kim, A.R., et al. (2021). A nonsense TMEM43 variant leads to disruption of connexin-linked function and autosomal dominant auditory neuropathy spectrum disorder. Proc. Natl. Acad. Sci. USA *118*, e2019681118.
16. Jumper, J., Evans, R., Pritzel, A., Green, T., Figurnov, M., Ronneberger, O., Tunyasuvunakool, K., Bates, R., Žídek, A., Potapenko, A., et al. (2021). Highly accurate protein structure prediction with AlphaFold. Nature *596*, 583–589.
17. Fagnani, E., Cocomazzi, P., Pellegrino, S., Tedeschi, G., Scalvini, F.G., Cossu, F., Da Vela, S., Aliverti, A., Mastrangelo, E., and Milani, M. (2024). CHCHD4 binding affects the active site of apoptosis inducing factor (AIF): Structural determinants for allosteric regulation. Structure *32*, 594–602.e4.
18. Thomas, L.W., Stephen, J.M., and Ashcroft, M. (2024). CHCHD4 regulates the expression of mitochondrial genes that are essential for tumour cell growth. Biochim. Biophys. Acta. Mol. Basis Dis. *1870*, 167282.
19. Hangen, E., Féraud, O., Lachkar, S., Mou, H., Doti, N., Fimia, G.M., Lam, N.V., Zhu, C., Godin, I., Muller, K., et al. (2015). Interaction between AIF and CHCHD4 Regulates Respiratory Chain Biogenesis. Mol. Cell *58*, 1001–1014.
20. Chang, H.C., Shapiro, J.S., Jiang, X., Senyei, G., Sato, T., Geier, J., Sawicki, K.T., and Ardehali, H. (2021). Augmenter of liver regeneration regulates cellular iron homeostasis by modulating mitochondrial transport of ATP-binding cassette B8. eLife *10*, e65158.
21. Reinhardt, C., Arena, G., Nedara, K., Edwards, R., Brenner, C., Tokatlidis, K., and Modjtahedi, N. (2020). AIF meets the CHCHD4/Mia40-dependent mitochondrial import pathway. Biochim. Biophys. Acta. Mol. Basis Dis. *1866*, 165746.
22. Habich, M., Salscheider, S.L., Murschall, L.M., Hoehne, M.N., Fischer, M., Schorn, F., Petrungaro, C., Ali, M., Erdogan, A.J., Abou-Eid, S., et al. (2019). Vectorial Import via a Metastable Disulfide-Linked Complex Allows for a Quality Control Step and Import by the Mitochondrial Disulfide Relay. Cell Rep. *26*, 759–774.e5.
23. Petrungaro, C., Zimmermann, K.M., Küttner, V., Fischer, M., Dengjel, J., Bogeski, I., and Riemer, J. (2015). The Ca(2+)-Dependent Release of the Mia40-Induced MICU1-MICU2

Dimer from MCU Regulates Mitochondrial Ca(2+) Uptake. Cell Metab. *22*, 721–733.

24. Rouault, T.A. (2019). The indispensable role of mammalian iron sulfur proteins in function and regulation of multiple diverse metabolic pathways. Biometals *32*, 343–353.
25. Wang, H., Shi, H., Rajan, M., Canarie, E.R., Hong, S., Simoneschi, D., Pagano, M., Bush, M.F., Stoll, S., Leibold, E.A., and Zheng, N. (2020). FBXL5 Regulates IRP2 Stability in Iron Homeostasis via an Oxygen-Responsive [2Fe2S] Cluster. Mol. Cell *78*, 31–41.e5.
26. Shaveta, G., Shi, J., Chow, V.T.K., and Song, J. (2010). Structural characterization reveals that viperin is a radical S-adenosyl-L-methionine (SAM) enzyme. Biochem. Biophys. Res. Commun. *391*, 1390–1395.
27. Terao, M., Garattini, E., Romão, M.J., and Leimkühler, S. (2020). Evolution, expression, and substrate specificities of aldehyde oxidase enzymes in eukaryotes. J. Biol. Chem. *295*, 5377–5389.
28. Ruiz-Pesini, E., Montoya, J., and Pacheu-Grau, D. (2021). Molecular Insights into Mitochondrial Protein Translocation and Human Disease. Genes *12*, 1031.

HGGA, Volume 7

Supplemental information

Biallelic variants in *CHCHD4* are associated with combined OXPHOS defect leading to mitochondrial disease

Matthieu Mantecon, Cerina Chhuon, Kevin Roger, Ida Chiara Guerrera, Christine Bole, Patrick Nitschke, Claire-Marie Dufeu-Bérat, Margaret Ashcroft, Robert W. Taylor, Nathalie Boddaert, and Agnès Rötig

Table of contents of Supplemental data

Figure S1

Figure S2

Figure S3

Figure S4

Table S1

Table S2 (supplied as an Excel file)

Supplemental Methods

- Cell culture
- Whole exome sequencing (WES)
- Whole-cell protein extracts
- SDS-PAGE and immunoblotting
- BN-PAGE
- CHCHD4 overexpression
- Proteomics analysis

Supplemental References

Figure S1

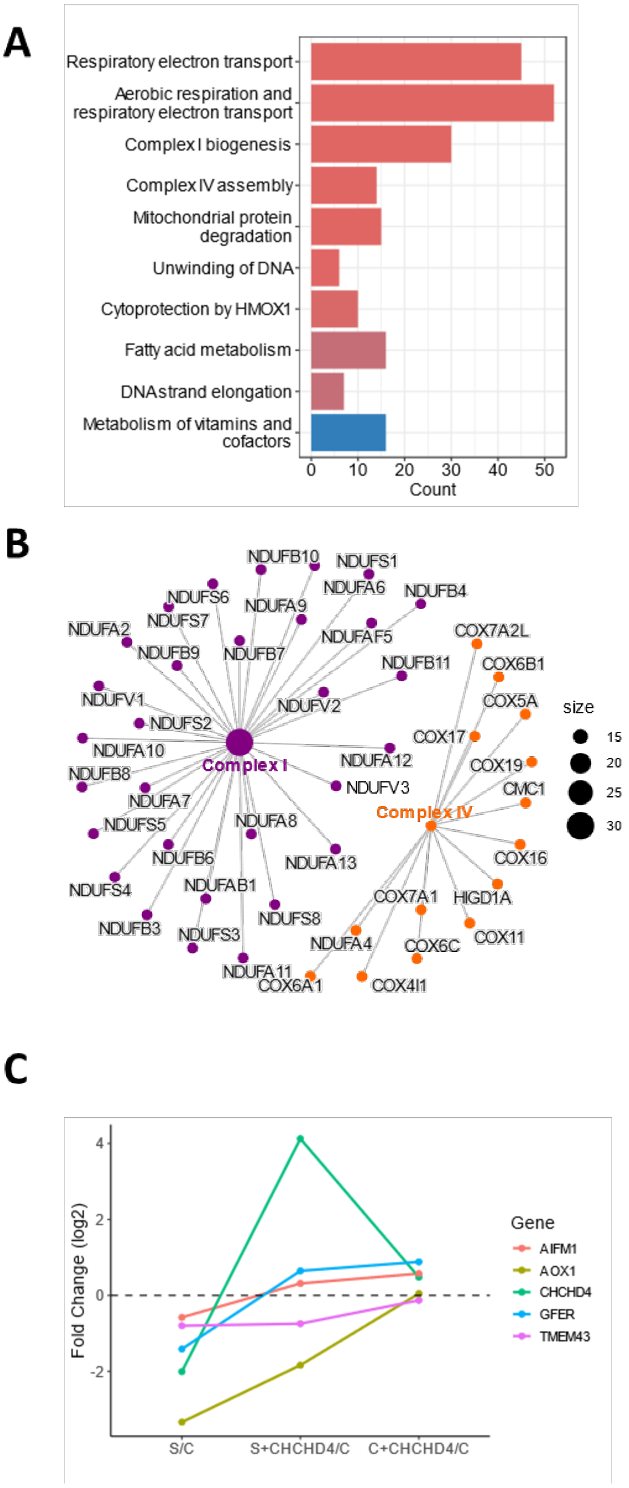

Proteomics analysis of subject (S) and control fibroblasts (C) transduced or not by wt *CHCHD4* cDNA. **A.** Reactome pathway analysis depicting the main dysregulated pathways in subject fibroblasts. **B.** Network of deregulated proteins from Complex I and Complex IV pathways in subject fibroblasts. **C.** CHCHD4 and other selected proteins levels in control fibroblasts expressed as fold change compared to control.

Figure S2

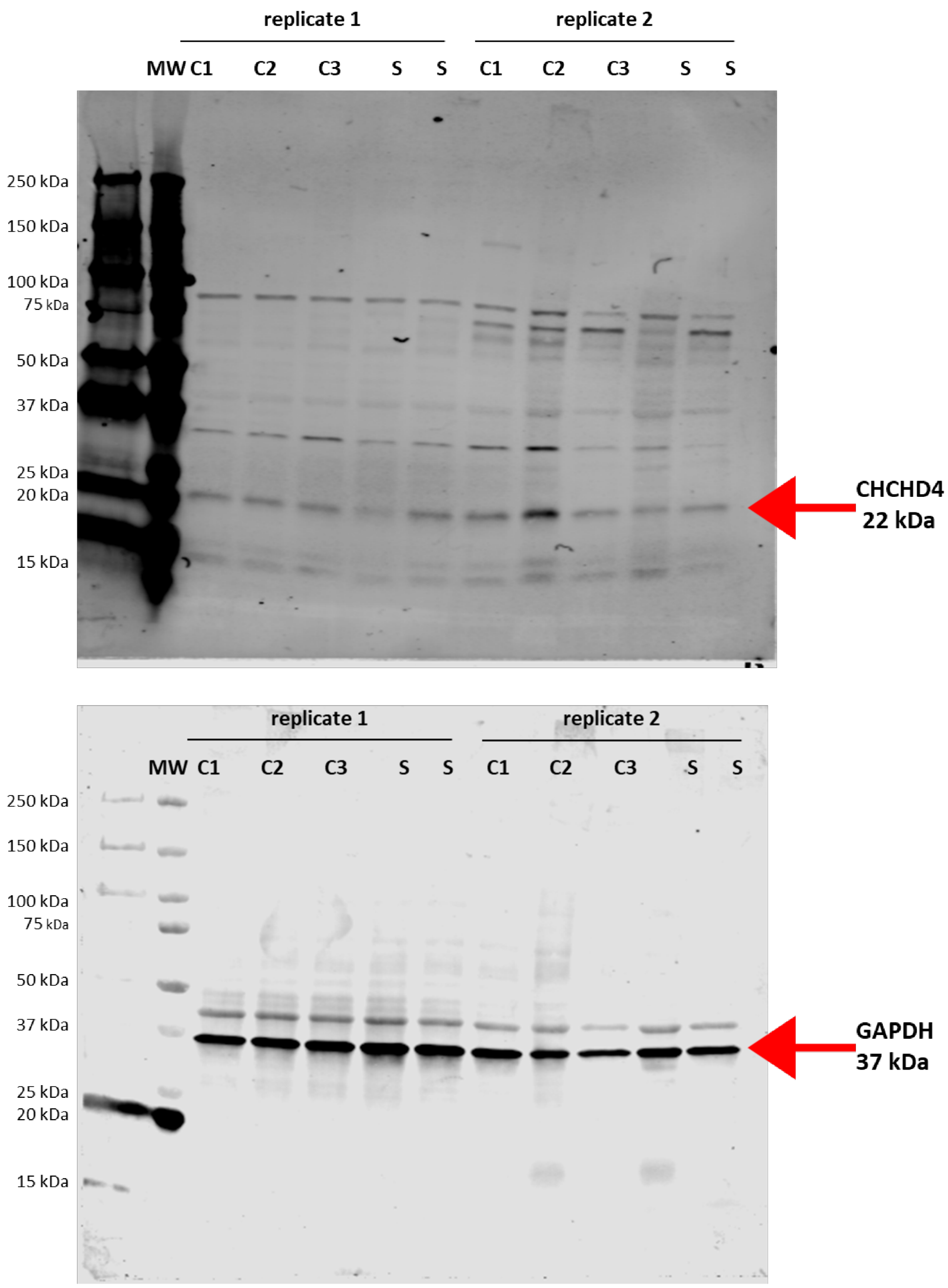

Biochemical investigation of fibroblasts. Full image of western blot analysis of CHCHD4 and GAPDH performed on total proteins of fibroblasts of subject (S) compared to 3 controls (C1, C2, C3). MW: molecular weight.

Figure S3

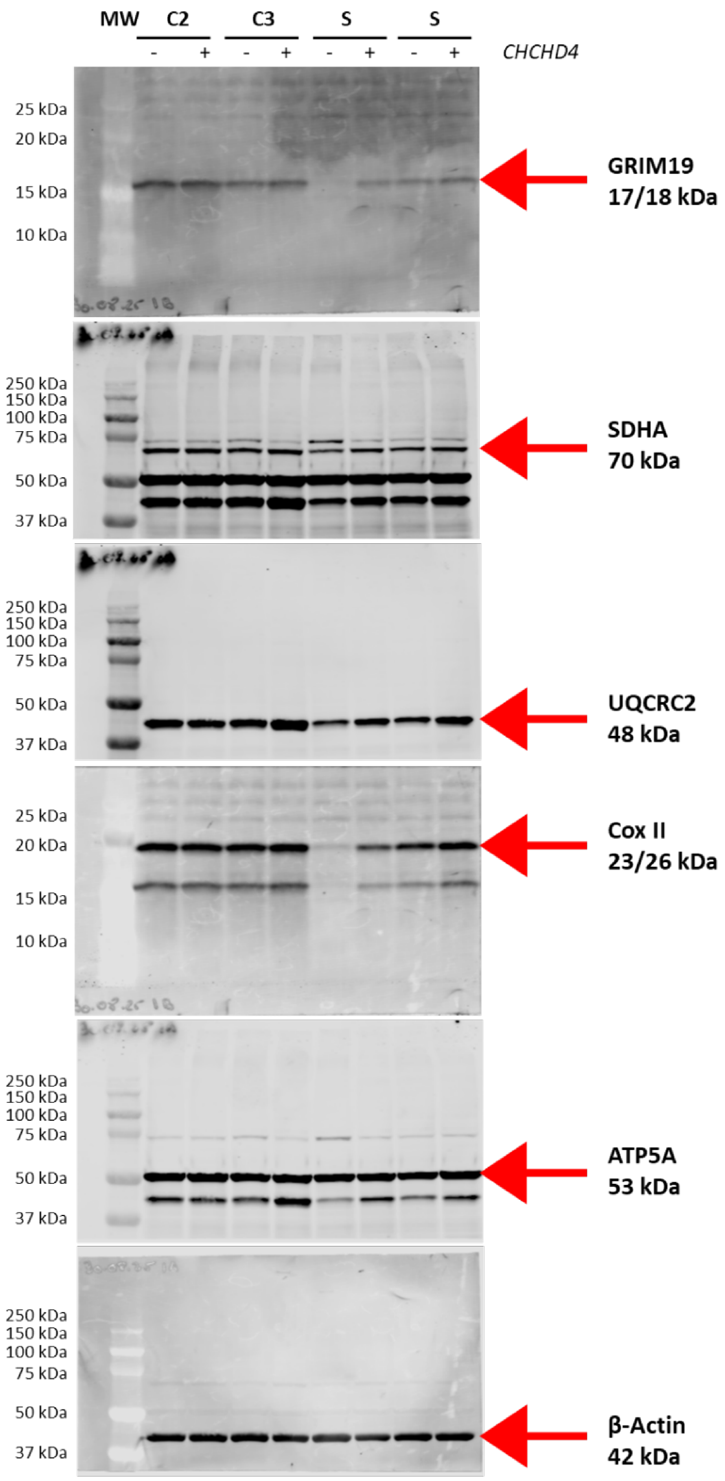

Biochemical investigation of fibroblasts. Full image of western blot analysis of OXPHOS subunits and β-actin in fibroblasts of subject and two controls (C2, C3) transduced with or without wt *CHCHD4* cDNA. MW: molecular weight.

Figure S4

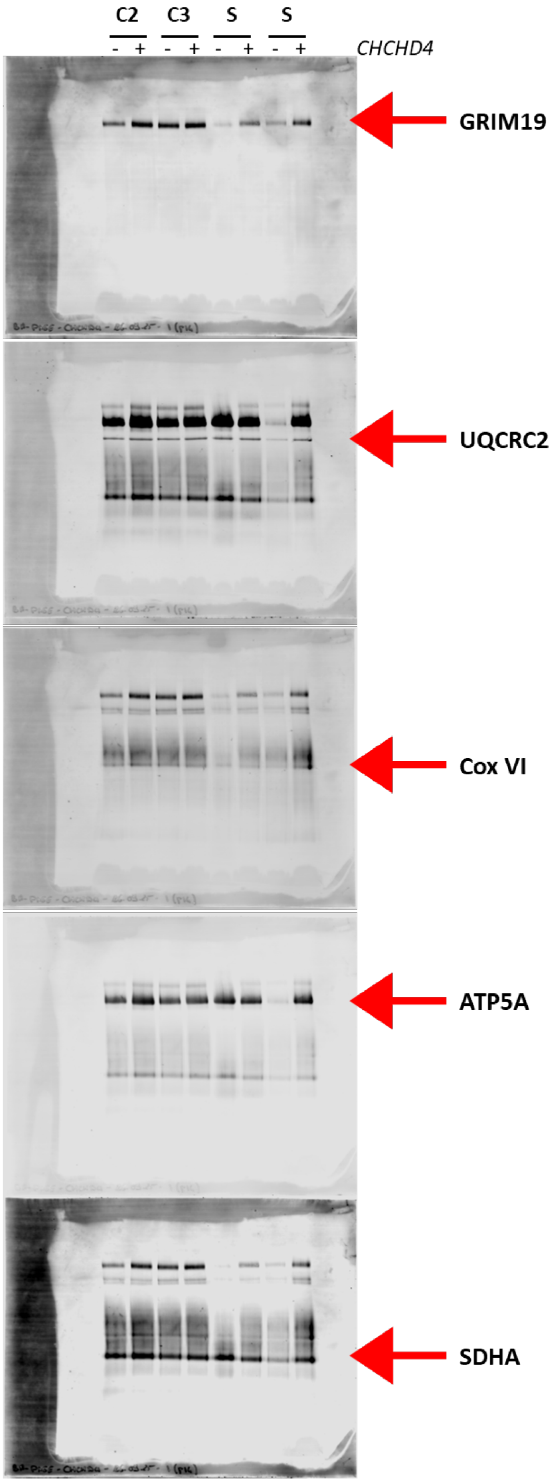

Biochemical investigation of fibroblasts. Full images of BN-PAGE on fibroblasts from subject (S) and two controls (C2, C3) transduced with or without wt *CHCHD4* cDNA.

HP:0001511	Intrauterine growth retardation
HP:0001943	Hypoglycemia
HP:0003128	Lactic acidosis
HP:0001410	Decreased liver function
HP:0001263	Global developmental delay
HP:0001298	Encephalopathy
HP:0012443	Abnormal brain morphology
HP:0025045	Abnormal brain lactate level by MRS

Table S1. Summary of clinical features of the subject Phenotypic findings of the subject mapped to HPO terms.

Table S2 is supplied as an Excel file

Table S2. Proteomic quantitative matrix and associated statistics combined with mitochondrial specific proteins from Mitocarta and over representation analysis of the down-regulated proteins from S_vs_S+CHCHD4 statistical analysis.

Supplemental Methods

Cell culture

Skin fibroblasts were grown in 5% CO2 at 37°C in Dulbecco's modified Eagle medium (DMEM, Gibco) supplemented with 4.5 g/L of D-glucose, 110 mg/L of sodium pyruvate, 10% fetal bovine serum (FBS, Gibco), and 100 U/mL of penicillin-streptomycin (Gibco).

Whole exome sequencing (WES)

Molecular genetic investigations were undertaken using EDTA-blood DNA samples. WES was performed on Subject as family-trio. WES data were interpreted according to American College of Medical Genetics (ACMG) guidelines1. The pathogenic variants were selected after filtering against known SNPs reported in dbSNP, 1000 Genomes, Exome Variant Server, in-house polymorphisms, and intergenic variants as previously described2.

Whole-cell protein extracts

Whole-cell protein extractions were carried out by suspending the cell pellets in RIPA buffer (ThermoFischer Scientific) supplemented with 1x Complete™ Protease Inhibitor Cocktail (Roche). Following an incubation on ice for 30 minutes the suspensions were centrifuged for 30 minutes at 16000 g in a cooled centrifuge. The supernatants were collected and stored at -20°C.

SDS-PAGE and immunoblotting

The protein concentration of the cell lysates was determined using BCA (ThermoFischer Scientific). 15 40 µg of whole-cell extracts, supplemented with Laemli buffer (Bio-Rad, Marnes-la-Coquette, France) and 100mM DTT (Merck), were fractionated through precast AnyKD Criterion TGX polyacrylamide gels (Bio-Rad) and blotted onto PVDF membranes (Bio-Rad). For analysis of the abundance of CHCHD4, samples were fractionated through AnyKD gels and immunoblotting was carried out using the following antisera: anti-CHCHD4 (HPA034688, Sigma Aldrich) and GAPDH (10494-1-AP, ProteinTech). OXPHOS proteins were fractionated through AnyKD gels and detected using the following antisera: anti-Grim19 (10986-1-AP, ProteinTech) for Complex I, anti-SDHA (ab14715, AbCam) for Complex II, 4 anti-UQCRC2 (ab14745, AbCam) for Complex III, anti-Cox II (55070-1-AP, ProteinTech) for Complex IV, anti-ATP5A (ab14748, AbCam) for Complex V and -anti-β-Actine conjugated with Alexa Fluor® 790 (ab184576, AbCam). IR Dye-conjugated (680RD or 800CW), secondary mouse or rabbit antisera were from Li-Cor Biosciences (Bad Homburg, Germany). Immunoblots were visualized with the Odyssey CLX infrared scanner (LI-COR Biosciences) and quantified using LI-COR's Image Studio.

BN-PAGE

For blue-native gel electrophoresis (BN-PAGE), mitoplasts were extracted from ~1x106 fibroblasts by incubation in 200 µl PBS containing 2 mg/ml Digitonin (Calbiochem) for 10 min on ice followed by dilution with 1 ml of PBS and centrifugation at 16000 x g for 10 min in a chilled centrifuge. The

mitoplast-containing pellets were washed once with PBS and resuspended in 60 µl ACBT buffer (1.5 M aminocaproic acid and 75 mM Bis-Tris (Sigma-Aldrich)) supplemented with 2% n-Dodecyl β-D maltoside (Sigma-Aldrich). After incubation on ice for 10 min, samples were centrifuged in a prechilled centrifuge at 16000 g for 30 min. Protein concentration in the extracts was determined using Bradford reagent (Sigma-Aldrich) and 10-15 µg of the extract were supplemented with Native PAGE sample buffer and Coomassie Brilliant Blue G-250 (ThermoFischer Scientific) prior to their fractionation through 4-16% NativePAGE Bis-Tris gel (ThermoFischer Scientific). Separated complexes were blotted onto a Immobilon-FL PVDF membrane (Millipore). OXPHOS complexes were detected using antibodies reactive against individual proteins from the five complexes: anti-Grim19 (10986-1-AP, ProteinTech) for complex I, anti-SDHA (ab14715, Abcam) for complex II, anti-UQCRC2 (ab147495, Abcam) for complex III, anti-COXIV (11242-1-AP, Proteintech) for complex IV and anti-ATP5A (Abcam, ab14748, Abcam) for complex V.

CHCHD4 overexpression

Fibroblasts grown in regular culture medium were transduced with human CHCHD4 cDNA cloned into the pD2109-CMV lentiviral vector (Atum), as previously described 3. Puromycin (Gibco) was added 72 hours after transduction at a final concentration of 5 µg/ml and selection was performed for 2 weeks during which media was changed every two days.

Proteomics analysis

5 S-TrapTM plate (Protifi, Hutington, USA) digestion was performed on 30 µg of cell lysates according to manufacturer's instructions. Briefly, samples were supplemented with 20% SDS to a final concentration of 5%, reduced with 20mM TCEP (Tris(2-carboxyethyl) phosphine hydrochloride) and alkylated with 50 mM chloracetamide (CAA) for 5 min at 95oC. Aqueous phosphoric acid was then added to a final concentration of 2.5% followed by the addition of S-Trap binding buffer (90% aqueous methanol, 100 mM TEAB, pH7.1). Mixtures were then loaded on S-Trap plate. Five washes were performed for thorough SDS elimination. Samples were digested with 1.5 µg of trypsin (Promega) at 47°C for 2 h. After elution, peptides were vacuum dried and resuspended in 2% ACN, 0.1% formic acid in HPLC-grade water prior to MS analysis. Peptides were resuspended in 30 µL of 2% ACN, 0.1% formic acid in HPLC-grade water and 300 ng were injected on an Evosep One system coupled to a timsTOF HT (Bruker Daltonics, Germany) mass spectrometer. The Evosep One system operated with the Whisper Zoom 40 Samples Per Day method using a 15 cm C18 Aurora Elite column (AUR3-15075C18-CSI, IonOpticks). The mobile phases comprised 0.1% FA as solution A and 0.1% FA/99.9% ACN as solution B. Mass-spectrometric data were acquired using the parallel accumulation serial fragmentation (PASEF) acquisition method in DIA (Data independent Analysis) mode with a 21-windows method using 25 Da windows covering the mobility ranges over a 475-1000 m/z range. The range of ion mobilities values from 0.85 to 1.27 V s/cm2 (1/k0). The total cycle time was set to 0.95 s. Data analysis was

performed using DIA-NN software (version 1.8.2). A search against the human UniProtKB/Swiss-Prot Homo sapiens database (downloaded the 12th of February, 2025, 20417 entries) was performed using library free workflow. For this purpose, “FASTA digest for library free search/library generation” and “Deep learning spectra, RTs and IMs prediction” options were checked for precursor ion generation. A maximum of 1 trypsin missed cleavages was allowed and the maximum variable modification was set to 2. Carbamidomethylation (Cys) was set as the fixed modification, whereas protein N-terminal methionine excision, methionine oxidation and N-terminal acetylation 6 were set as variable modifications. The peptide length range was set to 7–30 amino acids, precursor charge range 2–4, precursor m/z range 300–1300, and fragment ion m/z range 300–1300. To search the parent mass and fragment ions, accuracy was set to 10 ppm manually. The false discovery rates (FDRs) at the protein and peptide level were set to 1%. Match between runs was allowed. For the quantification strategy, Robust LC (high precision) was used as advised in the software documentation, whereas default settings were kept for the other algorithm parameters. Statistical and bioinformatic analysis were performed with MassDynamics 2.0 software available at https://massdynamics.com. All R figures was created using R (version 4.4) and RStudio (version 2025.09). The majority of the figures were created using ggplot2 (v3.5.2) embedded in the tidyverse (v2.0). In particular for Principal Component Analysis (PCA) and heatmap representation, the PCAtools (v2.18.0) and ComplexHeatmap R (v2.22) package were used, respectively. Over Representation Analysis using Cellular Component Gene Ontology (GO CC) and subsequent representation of the results was performed using ClusterProfiler (v4.14.6), enrichplot (v1.27.4) and org.Hs.eg.db (v3.20.0) R packages, respectively. Data have been deposited to the ProteomeXchange Consortium via the PRIDE4 partner repository with the dataset identifier PXD069027.

Supplemental References

1. Richards, S., Aziz, N., Bale, S., Bick, D., Das, S., Gastier-Foster, J., Grody, W.W., Hegde, M., Lyon, E., Spector, E., et al. (2015). Standards and guidelines for the interpretation of sequence variants: a joint consensus recommendation of the American College of Medical Genetics and Genomics and the Association for Molecular Pathology. Genet Med 17, 405-424. 2. Thompson, K., Bianchi, L., Rastelli, F., Piron-Prunier, F., Ayciriex, S., Besmond, C., Hubert, L., Barth, M., Barbosa, I.A., Deshpande, C., et al. (2022). Biallelic variants in TAMM41 are associated with low muscle cardiolipin levels, leading to neonatal mitochondrial disease. HGG Adv 3, 100097.
3. Gardeitchik, T., Mohamed, M., Ruzzenente, B., Karall, D., Guerrero-Castillo, S., Dalloyaux, D., van den Brand, M., van Kraaij, S., van Asbeck, E., Assouline, Z., et al. (2018). Bi-allelic Mutations in the Mitochondrial Ribosomal Protein MRPS2 Cause Sensorineural Hearing Loss, Hypoglycemia, and Multiple OXPHOS Complex Deficiencies. Am J Hum Genet 102, 685-695.
4. Perez-Riverol, Y., Bandla, C., Kundu, D.J., Kamatchinathan, S., Bai, J., Hewapathirana, S., John, N.S., Prakash, A., Walzer, M., Wang, S., et al. (2025). The PRIDE database at 20 years: 2025 update. Nucleic Acids Res 53, D543-D553.
